# Pharmaceutical Options for Triggering of Final Oocyte Maturation in ART

**DOI:** 10.1155/2014/580171

**Published:** 2014-07-15

**Authors:** Juan Carlos Castillo, Peter Humaidan, Rafael Bernabéu

**Affiliations:** ^1^Instituto Bernabeu, Avenida Albufereta 31, 03016 Alicante, Spain; ^2^The Fertility Clinic, Skive Regional Hospital, Faculty of Health, Aarhus University, 7800 Skive, Denmark; ^3^Faculty of Health, University of Southern Denmark, 5230 Odense M, Denmark

## Abstract

Since the pioneering days of *in vitro* fertilization, hCG has been the gold standard to induce final follicular maturation. We herein reviewed different pharmaceutical options for triggering of final oocyte maturation in ART. The new upcoming agent seems to be GnRHa with its potential advantages over hCG trigger. GnRHa triggering elicits a surge of gonadotropins resembling the natural midcycle surge of gonadotropins, without the prolonged action of hCG, resulting in the retrieval of more mature oocytes and a significant reduction in or elimination of OHSS as compared to hCG triggering. The induction of final follicular maturation using GnRHa represents a paradigm shift in the ovulation triggering concept in ART and, thus, a way to develop a safer IVF procedure. Kisspeptins are key central regulators of the neuroendocrine mechanisms of human reproduction, who have been shown to effectively elicit an LH surge and to induce final oocyte maturation in IVF cycles. This new trigger concept may, therefore, offer a completely new, “natural” pharmacological option for ovulation induction. Whether kisspeptins will be the future agent to trigger ovulation remains to be further explored.

## 1. Introduction and Background

Since the pioneering days of* in vitro* fertilization (IVF), human chorionic gonadotropin (hCG) has been the gold standard to induce final follicular maturation. As it is pharmacologically easily available for decades, hCG has been used as a surrogate for the natural midcycle luteinizing hormone (LH) surge. Due to the structural and biological similarities, hCG and LH bind to and activate the same receptor, the LH/hCG receptor [1]. An important difference, however, exists between the half-life of LH and hCG, whereas the half-life of LH is approximately 60 minutes [2], that of hCG exceeds 24 hours [3]. A sustained luteotropic activity induced by hCG is prone to cause undesired effects, notably, the release of vasoactive substances—primarily, vascular endothelial growth factor (VEGF)—through direct effects on the stimulated ovarian follicles. This may induce the occurrence of the most worrying side-effect of ovarian stimulation in IVF/ICSI cycles, namely, the ovarian hyperstimulation syndrome (OHSS).

**Figure 1:**
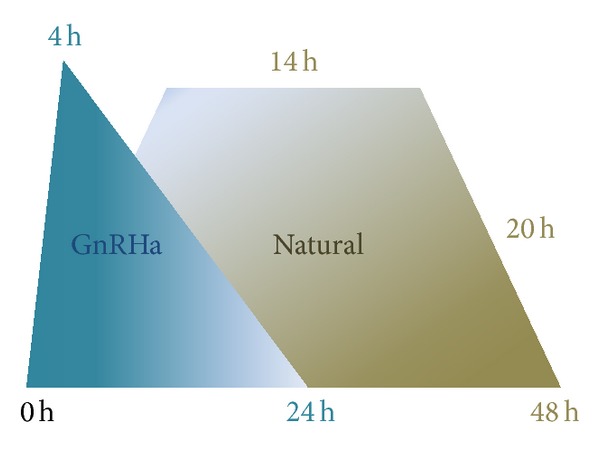
Differences in LH surge after GnRH-agonist triggering when compared with a natural cycle.

More than 30 years ago, Nakano et al. [4] described that it was possible to trigger an endogenous LH surge sufficient for induction of ovulation with a single injection of a gonadotropin-releasing hormone (GnRH) agonist (GnRHa). Unfortunately, this finding was soon underestimated, as GnRHa rapidly became the first line treatment to prevent premature luteinization, which indeed precluded the use of GnRHa to induce final follicular maturation. When the third generation GnRH antagonist was introduced into the market for use in ovarian stimulation protocols during the 1990's [5, 6] it became possible to trigger final oocyte maturation and ovulation with a single bolus of a GnRHa as an alternative to hCG. A particular property of the GnRH antagonist is its reversible effect, rapid action, and short duration, which allows the pituitary to remain “reactive” to the action of a single bolus of GnRHa for triggering ovulation. From a physiological point of view, a bolus of GnRHa displaces the GnRH antagonist from the receptor, which activates the receptor, inducing a release of follicle-stimulating hormone (FSH) in addition to LH (the “flare-up” effect), comparable to the natural midcycle surge of gonadotropins [4]. However, there are some important differences as the midcycle LH surge of the natural cycle is characterized by three phases and lasts for 48 hours [7], whereas the GnRHa induced surge consists of two phases, only: a short ascending limb (4 hours) and a long descending limb (20 hours), in total of 24–36 hours (Figure 1) [8]. Thus, the total amount of gonadotropins released during the surge is significantly reduced when GnRHa is used to trigger ovulation when compared with the natural cycle. The shorter duration of the endogenous LH surge induced by GnRHa triggering seems to play a key role for the reduced risk of OHSS development when GnRHa is used to trigger final oocyte maturation [9].

Many studies show that GnRH agonists are as effective as hCG to induce an adequate final follicular maturation and at the same time to prevent OHSS [10]. However, another possible advantage of GnRHa for triggering of final oocyte maturation is the simultaneous induction of a FSH surge comparable to the surge of the natural cycle. The specific role of the midcycle FSH surge that accompanies the LH midcycle surge during the natural menstrual cycle is not fully understood, but FSH presumably acts synergistically with LH to promote the optimal environment for final oocyte maturation and ovulation. In general, FSH is known to promote formation of LH receptors in luteinizing granulosa cells and seems to promote oocyte nuclear maturation and cumulus expansion [11, 12]. FSH also has a role in maintaining gap junctions between the oocyte and cumulus cells and, thus, may have an important role in signaling pathways [13]. Interestingly, several studies including two RCTs reported the retrieval of more mature oocytes after GnRHa trigger, which might be attributed to the presence of a surge of FSH as well as LH [14, 15].

GnRHa preparations are known to vary in their relative potencies; nonetheless, all of them seem to perform adequately in clinical practice. Thus, Parneix et al. [16] studied a variety of protocols, using different GnRHa types administered at different doses and intervals. It appeared that no regimen was superior to the other and all protocols examined induced LH/FSH surge and subsequent successful ovulation.

Currently, various short-acting GnRHa preparations are used as trigger agents. Most recent studies have used single doses of the following types of GnRHa: either triptorelin 0.2 mg [17], buserelin 0.5 mg [18], leuprolide acetate 1 mg [19], or leuprolide acetate 1.5 mg [20]. The timing of the oocyte pickup after GnRHa administration has been reported to be the same as after hCG triggering (34–36 hours).

Follicular phase cotreatments with GnRH agonist and hCG-based induction of the final stages of oocyte maturation before oocyte retrieval have been the standard of care in IVF clinical practice over the last 30 years. However, after the widespread use of GnRH antagonist administration, alternative approaches for the induction of oocyte maturation have received increasing attention in recent years. GnRH-agonist triggering opens the door for a paradigm shift in the ovulation triggering concept in ART underlying the importance of developing and optimizing ovarian stimulation protocols for an effective, physiologic, and safe management of final oocyte maturation in ART.

## 2. GnRHa Trigger and Oocyte/Embryo Quality: The Oocyte Donor Model

Compelling evidence concurs to indicate that the use of GnRHa triggering for final oocyte maturation in oocyte donors apart from eliminating the risk of any clinically significant OHSS secures the retrieval of oocytes of a quality similar to that seen after hCG trigger and, importantly, with a similar reproductive outcome in the recipient.

Large oocyte donor database retrospective studies [17] and a number of methodologically more appropriate randomized clinical trials [21–23] found no significant differences in the number of retrieved oocytes (total and mature), fertilization rates, embryo quality, and pregnancy rates, indicating that GnRHa trigger and hCG trigger provided equivalent outcomes in the recipients. Importantly, OHSS was not reported after GnRHa triggering, whereas the OHSS incidence after hCG triggering was between 4 and 17% [10].

In an oocyte donor population, other additional benefits may help to substantially decrease the treatment burden of the patient, including a shorter duration of the luteal phase (4–6 days), a reduced ovarian volume [18], diminished abdominal distension, and avoidance of estradiol monitoring during stimulation [24]. These factors simplify the clinical management of the oocyte donation treatment for the donor as well as for the clinician.

## 3. The Luteal Phase after GnRH-Agonist Triggering of Ovulation 

Previous randomized controlled trials [25, 26] showed that the use of GnRHa for triggering ovulation was associated with a markedly decreased ongoing clinical pregnancy rate and a high rate of early pregnancy loss, presumably attributed to a luteal phase insufficiency despite standard supplementation with vaginal progesterone and estradiol. More recently, several studies now report a luteal phase rescue after modified luteal phase support, resulting in a reproductive outcome comparable to that seen after hCG triggering [10]. Thus, intensive luteal support with IM progesterone and estradiol, only, after GnRHa trigger in some reports does not result in low ongoing pregnancy rates [27, 28]. Others proposed to overcome the luteal phase problems reported following GnRHa triggering by adding minimal amounts of hCG for luteal support either in the form of one bolus of 1500 UI of hCG [14, 18, 29] or repeated boluses (250–500 IU) of hCG [20] or by the addition of recombinant LH [30].

The most plausible reason for the luteal phase insufficiency seen after ovarian stimulation with gonadotropins is the combination of a multifollicular development and triggering of ovulation with hCG which, with its prolonged half-life, results in supraphysiological levels of progesterone and estradiol. The supraphysiological steroid levels directly inhibit the LH secretion from the pituitary [31, 32], resulting in luteal LH insufficiency [33] and subsequent corpus luteum demise. Thus, luteal phase support with progesterone, either vaginally or intramuscularly, remains mandatory in all IVF protocols [34, 35].

In a “proof of concept” study, Kol et al. [36] described a novel protocol in which final oocyte maturation was induced with a bolus of GnRHa followed by an hCG-based luteal support, without any exogenous luteal progesterone or estradiol supplementation. Thus, the luteal phase was supported with two boluses of hCG, only. The patients included in the study developed ≤12 follicles on the day of trigger and a high ongoing clinical pregnancy rate was reported. Clearly, the findings of this study need to be corroborated in a future large study; however, the concept introduces a simple and patient friendly luteal phase support, avoiding vaginal applications, discharge, and painful progesterone injections [36].

## 4. OHSS after GnRHa Triggering

The main reason to use GnRHa trigger as a substitute for hCG trigger is the expected total elimination of any clinically relevant (moderate/severe) OHSS. In fact, in the largest randomized, controlled trial published to date in a population at high risk of OHSS (follicle count 15–25 follicles) [18], not a single case of OHSS was described, despite the use of a low-dose hCG rescue protocol followed by fresh embryo transfer. Importantly, the reproductive outcome was comparable to that of hCG trigger. Moreover, a number of clinical trials in the oocyte donor population [22, 23, 37] reported a complete elimination of OHSS after GnRHa triggering.

However, following the increased usage of GnRHa trigger worldwide, recent publications have challenged the previous conclusions. Thus, Seyhan et al. [38] presented a case series of 23 IVF patients at high risk of OHSS, who received the low-dose hCG rescue protocol as described by Humaidan et al. [18, 29, 39]. The authors reported a 22% early onset severe OHSS rate. However, an in-detail look at the patient characteristics reveals the inclusion of extreme high responder patients with up to 50 or 65 oocytes. Moreover, 8 of these high risk patients in addition received either 2 or 3 embryos for transfer, which further increases the risk of subsequent late onset OHSS. An accompanying editor's comment and a prompt letter to the editor by Humaidan et al. [40] raised surprise and concerns regarding the application of the new protocol in candidates, clearly not suitable to receive 1500 UI hCG for luteal support. Currently, available data suggest that GnRHa trigger followed by a modified low-dose early luteal hCG support provides the normoresponder patient and the moderate-high OHSS risk patient (up to 25 follicles >11 mm) with the opportunity to proceed to fresh embryo transfer with good ongoing pregnancy rates and a very low OHSS risk. In contrast, until prospective studies help fine-tune the minimal hCG activity needed for luteal phase support after GnRHa trigger, patients with a higher OHSS risk (>25 follicles) currently benefit from a freeze-all strategy. In conclusion, GnRHa trigger and modified luteal support with one bolus of hCG should be used with caution in extremely high responder patients.

As a means to completely prevent the risk of OHSS development in OHSS risk patients, a segmentation of the IVF treatment has recently been proposed [41]. The so-called “OHSS free clinic” [42] defines a strategy in which ovarian stimulation and trigger is separated from the embryo transfer. Thus, IVF/ICSI patients with GnRH antagonist cotreatment have final follicular maturation using a bolus of GnRHa followed by a total freeze of all embryos for transfer in subsequent cycles. According to the authors, this strategy would completely eliminate early as well as late onset OHSS. However, in a recent publication [43], two patients following this procedure developed severe OHSS requiring hospitalization and ascites drainage. Interestingly, in these two cases—one IVF patient and one oocyte donor—the GnRHa trigger apparently did not induce the usual luteal phase insufficiency associated with GnRHa trigger as patients menstruated as late as 12 and 14 days after the oocyte retrieval. Although, the exact etiology of these cases remains unknown, the authors speculated whether GnRH receptor or FSH or LH receptor gene mutations led to a prolonged LH/FSH rise or abnormal activation of LH/FSH receptors, explaining the OHSS development and the long duration of the luteal phase.

## 5. Failure of GnRHa Triggering of Final Follicular Maturation

The “empty follicle” syndrome (EFS) is characterized by the lack of retrieval of oocytes from apparently normally growing ovarian follicles with normal estradiol levels after ovarian stimulation. This quite rare and frustrating condition has an uncertain etiology; most cases of EFS after either hCG or GnRHa triggering are related to human error, and, thus, a meticulous counseling and instruction of the patient prior to oocyte retrieval is of outmost importance. However, as the pituitary is the target organ for GnRHa, certain forms of pituitary dysfunctions, such as partial hypothalamic disorders and/or profound (temporary/permanent) pituitary suppression, might be responsible for these outcomes in GnRHa triggered cycles [44].

Interestingly, some cases of EFS after hCG triggering are solved by changing the trigger agent to GnRH agonist in GnRH antagonist cycles [45]. In these cases, one might assume that a more physiological LH plus FSH surge may promote an adequate final follicular maturation, preventing the occurrence of EFS. As previously mentioned, in contrast to hCG triggering, the action of a bolus of GnRHa is indirect via the endogenous release of LH and FSH from the pituitary after binding to and activation of the GnRH receptor. Thus, EFS after GnRHa triggering may represent a different pathology as compared to EFS after hCG triggering. Importantly, a recent large database analysis showed that the incidence of EFS seems to be similar regardless of whether GnRHa (3.5%) or hCG (3.1%) triggering is used for final oocyte maturation [44].

## 6. GnRH-Agonist Triggering: Concluding Remarks

GnRHa trigger is currently used worldwide and its use is steeply increasing. GnRHa trigger is now part of the current standard of care [46], and although GnRHa trigger is principally used to avoid the risk of OHSS development, the potential advantages and clinical applications of GnRHa trigger are numerous. Future trials are needed to explore the minimal amount of exogenous hCG necessary for luteal phase support after GnRHa trigger to avoid OHSS and at the same time to secure high ongoing pregnancy rates.

## 7. Kisspeptins for Final Follicular Maturation in the Horizon

Kisspeptins (KP) involve a group of recently discovered peptide hormones, which play a key role in the neuroendocrine regulation of human reproduction [47]. After the discovery of GnRH in the early 1970's [48], researchers started looking for the anatomical location of the mechanism generating GnRH pulses. The discovery of KP neurons in the hypothalamus has provided a clue to the possible location of the GnRH pulse generator; these neurons located in the rostral preoptic area and the infundibular nucleus in the human hypothalamus [48] seem to play a central role in the generation of GnRH pulses in mammalian species. KP, a hypothalamic peptide coded by the KiSS1 gene, has a fundamental role in control of the gonadal axis and is now recognized as an important regulator of the onset of puberty, the regulation of sex hormone-mediated secretion of gonadotropins, and the control of fertility [49]. KP signals directly to the GnRH neurons through actions on the KP receptor to release GnRH into the portal circulation, which in turn stimulates the secretion of both LH and FSH from the gonadotrophs of the anterior pituitary, although the effect on the former is more marked [50].

Kisspeptins are potent stimulators of the hypothalamic-pituitary-gonadal axis. The knowledge of the stimulatory effect of exogenous KP on the secretion of LH at the time of ovulation in humans derives from preliminary experimental investigations; however, recent data support a potential role for KP in generating the ovulatory LH surge in humans. Thus, Jayasena et al. [51] showed that exogenous KP was able to induce a 3-4-fold increase in LH secretion when administered in the periovulatory phase and the repeated twice-daily administration of KP shortened the menstrual cycle and advanced the onset of the LH peak in healthy women [52]. Although much remains to be learned about the role of kisspeptins in the control of ovulation and its actions at central and/or ovarian level, the results of the preliminary studies pave the way for the potential use of KP as agents, inducing a physiological final follicular maturation.

Very recently, in IVF cycles, Abbara et al. [53] described that KP were able to effectively elicit an LH surge to induce final oocyte maturation with subsequent successful achievement of live births. This new trigger agent may, therefore, offer a completely new, “natural” pharmacological option for ovulation induction in ART. Importantly, the risk of OHSS might be eliminated.

## 8. Conclusion

We herein reviewed different pharmaceutical options for triggering of final oocyte maturation in ART.  Table 1 summarizes current knowledge and recommendations. Although hCG for decades has been the gold standard for final oocyte maturation, the new upcoming agent seems to be GnRHa with its potential advantages over hCG trigger, mainly in terms of OHSS reduction. Over the years, the luteal phase support after GnRHa trigger has been refined to a degree where the reproductive outcome is similar to that seen after hCG trigger. Moreover, GnRHa trigger opens the possibility to “tailor” the luteal phase support according to the ovarian response to stimulation. Importantly, in OHSS high risk patients, GnRHa trigger may be safely performed, followed by a “freeze-all” strategy with minimal risk of OHSS development and a high cumulative pregnancy rate in subsequent frozen embryo transfer cycles. Whether kisspeptins will be the future agent to trigger ovulation remains to be explored in large clinical trials.

## Figures and Tables

**Table 1:** Pharmaceutical options for the triggering of final oocyte maturation in ART: summary and recommendations.

Subject	Current knowledge	Recommendations
GnRHa trigger and oocyte/embryo quality: the oocyte donor model	No significant differences in the number of retrieved oocytes (total and mature), fertilization rates, embryo quality, and pregnancy rates in recipients	First line treatment in egg donors
	Substantial decrease in the treatment burden of the egg donor	

The luteal phase after GnRH-agonist triggering of ovulation	GnRH-agonist triggering is associated with luteal phase insufficiency despite the standard supplementation with vaginal progesterone and estradiol	Luteal phase rescue protocols:
		1500 IU hCG, 35 h after GnRHa trigger∗
		IM prog + E2 patches adjusted according to serum levels∗
		Repeated bolus of 500 IU hCG
		Repeated bolus of rec-LH
		Freeze-all strategy

OHSS after GnRHa triggering	OHSS cases described in extremely high responders who received the 1500 IU hCG rescue protocol	GnRHa trigger and modified luteal support with one bolus of hCG should be used with caution in extremely high responder patients
		Patients with a higher OHSS risk (>25 follicles) currently benefit from a freeze-all strategy
	Two OHSS cases reported after GnRHa triggering without any type of luteal phase support	Rare event of unknown etiology
		GnRH, FSH, or LH receptor gene mutations presumably involved

Failure of GnRHa triggering of final follicular maturation	A recent large database analysis showed that the incidence of EFS seems to be similar regardless of whether GnRHa (3.5%) or hCG (3.1%) triggering is used for final oocyte maturation	Certain forms of pituitary dysfunctions might be responsible for these outcomes in GnRHa triggered cycles
		Most cases of EFS are related to human error, and, thus, a meticulous counseling and instruction of the patient prior to oocyte retrieval is of outmost importance

Kisspeptins (KP) for final follicular maturation in the horizon	KP are potent stimulators of the hypothalamic-pituitary-gonadal axis	Much remains to be learned about the role of KP in the control of ovulation
	KP signals directly to the GnRH neurons, which in turn stimulates the secretion of both LH and FSH from the anterior pituitary that is able to induce a physiological final follicular maturation	The promising results of a preliminary study need to be further explored in large clinical trials

*Supported by large RCTs.
